# Trends in socioeconomic inequalities in cancer mortality in Barcelona: 1992–2003

**DOI:** 10.1186/1471-2458-9-35

**Published:** 2009-01-23

**Authors:** Rosa Puigpinós, Carme Borrell, José Leopoldo Ferreira Antunes, Enric Azlor, M Isabel Pasarín, Gemma Serral, Mariona Pons-Vigués, Maica Rodríguez-Sanz, Esteve Fernández

**Affiliations:** 1 Servei de Sistemes d'Informació Sanitaria, Agència de Salut Pública de Barcelona, Barcelona, Spain; 2 CIBER Epidemiologia y Salud Pública (CIBERESP), Barcelona, Spain; 3 Departament de Salut Pública, Facultat de Medicina. Universitat de Barcelona, Barcelona, Spain; 4 Departament de Ciències Experimentals i de la Salut, Universitat Pompeu Fabra., Barcelona, Spain; 5 School of Dentistry, Universidade de São Paulo, São Paulo, Brazil; 6 Tobacco Control and Research Unit, Institut6 Català d'Oncologia (ICO-IDIBELL), l'Hospitalet de lobregat (Barcelona), Spain; 7 Department of Clinical Sciences, Campus of Bellvitge, Universitat de Barcelona, l'Hospitalet de Llobregat (Barcelona), Spain

## Abstract

**Background:**

The objective of this study was to assess trends in cancer mortality by educational level in Barcelona from 1992 to 2003.

**Methods:**

The study population comprised Barcelona inhabitants aged 20 years or older. Data on cancer deaths were supplied by the system of information on mortality. Educational level was obtained from the municipal census. Age-standardized rates by educational level were calculated. We also fitted Poisson regression models to estimate the relative index of inequality (RII) and the Slope Index of Inequalities (SII). All were calculated for each sex and period (1992–1994, 1995–1997, 1998–2000, and 2001–2003).

**Results:**

Cancer mortality was higher in men and women with lower educational level throughout the study period. Less-schooled men had higher mortality by stomach, mouth and pharynx, oesophagus, larynx and lung cancer. In women, there were educational inequalities for cervix uteri, liver and colon cancer. Inequalities of overall and specific types of cancer mortality remained stable in Barcelona; although a slight reduction was observed for some cancers.

**Conclusion:**

This study has identified those cancer types presenting the greatest inequalities between men and women in recent years and shown that in Barcelona there is a stable trend in inequalities in the burden of cancer.

## Background

Although cancer mortality in the US and the European Union has levelled off or decreased since the 1980s, cancer mortality is on the increase in developing countries [1-4]. In fact, inequalities in the burden of cancer have been reported both between and within countries [5-8]. Different conceptual models link social factors and health outcomes and also include aspects related with differential access to treatment and prevention, and the exposure to cancer risk factors. The interplay of these dimensions would explain inequalities in cancer mortality between socioeconomic and ethnic groups, and differential survival rates for the most frequent tumours in men and women[9]. Overall, cancer inequalities are widening in part due to unequal access to the best diagnostic and therapeutic resources available[10].

In Barcelona, cancer mortality ranks higher for disadvantaged social classes [11,12]. Men with low educational level present higher mortality rates for mouth, pharynx, oesophagus, stomach and lung cancer; in women, inequalities are higher for cervix and corpus uteri cancer. Socioeconomic inequalities have also been reported to affect major risk factors (tobacco, alcohol, dietary patterns, physical activity)[13]; and inequalities in the burden of cancer are more prevalent among people aged 65 years or older [14]. The assessment of cancer mortality by socio-economic level allows health services to evaluate hypotheses on its determinants, and to improve programmes and interventions[15]. However, few studies have systematically assessed socio-economic inequalities in trends of cancer mortality, and there are no in-depth studies dealing simultaneously with many different cancer sites. Therefore, the objective of this study was to assess trends and the magnitude of inequalities in cancer mortality by educational level in the city of Barcelona, over the period 1992–2003.

## Methods

### Design and study population

The study population consists of persons aged 20 years and older, resident in Barcelona during the period 1992–2003. The sources of information were the mortality register and the municipal censuses. The mortality register of Barcelona is based on information corresponding to all death certificates of residents of Barcelona and is maintained jointly by the *Instituto Nacional de Estadística *(Spanish Institute of Statistics) and the Government of Catalonia ; it is highly exhaustive[16]. The educational level of the deceased was obtained through the municipal census, which includes socio-demographic variables of the population of Barcelona, by means of a record linkage between the register of mortality and the municipal census of Barcelona (complete for 94.7% of deaths). This is a confidential probabilistic linkage based on the name, surname and date of birth of the deceased [17].

Municipal censuses performed in Barcelona in 1991, 1996 and 2003 provided primary information on the population, stratified by age, sex and level of education, for the assessment of death rates. For intercensal years, the population was estimated by the method of geometric progression [18]. Every resident in Barcelona is registered in the municipal census, which is continually updated with information on migration, births and deaths. The Barcelona City Census is an administrative registry not subject to statistical secrecy.

### Study variable and covariates

The underlying cause of death was coded using the International Classification of Diseases (ICD) 9^th ^revision until 1999, and ICD 10^th ^revision thereafter. A previous study in five Autonomous Communities of Spain (Catalonia being one of them) found good agreement between ICD-9 and ICD-10 on the leading causes of death in Spain, particularly in the cancer-related causes of death [19]. The cancer sites included in this study are shown in Tables 1 and 2.

**Table 1:** Distribution of deaths among adults men (≥ 20 years) by cancer site and educational level.

**Cancer site**	**ICD-9 (1)**	**ICD-10 (2)**	**1992–94** **%**	**1995–97** **%**	**1998–00** **%**	**2001–03** **%**
**Mortality by cancer**			(n = 7721)	(n = 8016)	(n = 7892)	(n = 7781)

Stomach	151	C16	5.8	5.4	4.8	4.7
Colon	153	C18	7.8	8.6	9.6	9.2
Rectum	154	C19–C21	2.5	2.6	3.1	2.9
Larynx	161	C32	2.8	2.8	2.3	2.4
Lung	162	C33, C34	28.0	27.8	27.3	27.2
Mouth and pharynx	140–149	C00–C14	3.7	3.2	3.1	2.6
Oesophagus	150	C15	2.7	2.6	2.4	2.3
Liver	155	C22	6.4	6.2	6.1	5.2
Pancreas	157	C25	3.2	4.0	4.0	4.4
Kidney	189	C64–C66, C68	2.0	1.7	1.8	1.9
Brain (central nervous system)	191–192	C70–C72	1.7	1.8	1.9	2.4
Non-Hodgkin lymphoma	200. 203	C82–C85. C96	2.1	2.3	2.4	2.8
Leukaemia	204–208	C91–C95	2.5	2.5	2.5	3.3
Gallbladder	156	C23–C24	6.2	5.7	5.3	6.1
Prostate	185	C61	8.8	9.2	8.9	7.7
Tumours of ill-defined sites	195–199	C76–C80. C97*	5.3	5.6	5.9	6.5
Other tumours			8.5	8.1	8.6	8.4

**Educational level**						

No education			35.2	31.0	27.4	24.9
Incomplete primary education			33.6	34.0	35.9	35.2
Primary education			11.2	12.4	13.1	13.6
Secondary education			8.7	10.2	10.5	11.4
University education			11.3	12.5	13.3	14.8

**Population at risk**			(n = 1.676.468)	(n = 1.683.111)	(n = 1.742.299)	(n = 1.848.941)

No education			14.4	12.3	10.4	9.0
Incomplete primary education			26.5	25.2	25.5	25.7
Primary education			18.7	19.6	18.7	17.8
Secondary education			22.0	24.3	25.7	26.8
University education			18.3	18.6	19.6	20.6

TOTAL			100.0	100.0	100.0	100.0

Distribution of population at risk by educational level. Barcelona, 1992–2003.

Deaths without information on educational level have been excluded (1.528 men and 1.430 women)

(1) ICD-9: International Classification of Diseases. ninth revision.

(2) ICD-10: International Classification of Diseases. tenth revision.

n: number of cases.

**Table 2:** Distribution of deaths among adults women (≥ 20 years) by cancer site and educational level. Distribution of population at risk by educational level. Barcelona. 1992–2003.

**Cancer site**	**ICD-9 (1)**	**ICD-10 (2)**	**1992–94** **%**	**1995–97** **%**	**1998–00** **%**	**2001–03** **%**
**Mortality by cancer**			(n = 5160)	(n = 5350)	(n = 5287)	(n = 5438)

Stomach	151	C16	6.6	5.7	5.5	5.0
Colon	153	C18	11.6	11.4	12.7	11.5
Rectum	154	C19–C21	3.3	3.4	3.6	3.2
Lung	162	C33. C34	5.0	5.8	6.3	7.6
Mouth and pharynx	140–149	C00–C14	0.9	1.2	1.5	1.1
Liver	155	C22	5.4	5.4	5.0	4.9
Pancreas	157	C25	4.2	5.3	5.7	5.7
Kidney	189	C64–C66. C68	1.2	1.6	1.2	1.5
Brain (central nervous system)	191–192	C70–C72	2.3	2.5	2.5	2.6
Non-Hodgkin lymphoma	200. 203	C82–C85. C96	3.2	3.3	3.3	4.0
Leukaemia	204–208	C91–C95	3.4	3.3	3.3	4.2
Gallbladder	156	C23–C24	2.7	2.8	1.9	1.9
Breast	174	C50	20.1	17.9	17.5	16.4
Cervix uteri	180	C53	1.7	1.7	1.8	1.6
Corpus uteri	179. 182	C54. C55	3.5	3.2	3.5	2.8
Ovary and other female genital	183	C56–C57	4.4	4.5	4.1	4.8
Tumours of ill-defined sites	195–199	C76–C80. C97*	6.7	6.8	6.5	7.2
Other tumours			13.8	14.2	14.1	14.0

**Educational level (4)**						

No education			45.6	42.6	37.6	33.0
Incomplete primary education			34.1	35.7	37.2	38.4
Primary education			8.9	9.1	10.6	11.6
Secondary education			4.9	5.3	6.7	7.3
University education			6.6	7.3	7.9	9.7

**Population at risk**			(n = 1,676,468)	(n = 1,683,111)	(n = 1,742,299)	(n = 1,848,941)

No education			14.4	12.3	10.4	9.0
Incomplete primary education			26.5	25.2	25.5	25.7
Primary education			18.7	19.6	18.7	17.8
Secondary education			22.0	24.3	25.7	26.8
University education			18.3	18.6	19.6	20.6

TOTAL			100.0	100.0	100.0	100.0

Deaths without information on educational level have been excluded (1.528 men and 1.430 women).

(1) ICD-9: International Classification of Diseases. ninth revision.

(2) ICD-10: International Classification of Diseases. tenth revision.

n: number of cases.

The study covariates were sex, age (categorised into five-year age-groups: 20–24 years, 25–29 years, etc.) and educational level categorized in five groups as 'No education' (illiterate or persons with 0–4 years of schooling); 'Incomplete primary education' (subjects with uncompleted elementary education or 5–6 years of schooling; 'Primary education' (subjects with complete primary education or 7–9 years of schooling); 'Secondary education' (subjects with high school or 10–14 years of schooling); and 'University education' (subjects with University degree or postgraduate studies, corresponding to 15 years or more of schooling).

### Data analysis

Analyses were performed separately for men and women [20]. For descriptive and analytical purposes, deaths were grouped into four periods (1992–1994, 1995–1997, 1998–2000, and 2001–2003) in order to ensure sufficient numbers of deaths in each period. Age-standardized mortality rates for each three-year period were calculated by the direct method and using the mid-period population of Barcelona (1996) as the reference for standardization.

Poisson regression models [21] were fitted to obtain the relative index of inequality (RII), and corresponding 95% confidence intervals, for educational level, adjusting for age, in each period. The outcome variable was the log-transformed death rate for each type of cancer; the covariate was educational level, assessed parametrically (five values scaled between 0 and 1 for each category), and controlled by age. Population was introduced as an offset. The RII can be interpreted as the ratio between the death rates of the highest and the lowest educational levels[22] and has a interpretation similar to a risk ratio or relative risk.

In order to assess trends of the RII, deaths from 1992 to 2003 were pooled together to fit multivariate Poisson regression models with educational level, period and age as independent variables, as well as the interaction between educational level and period. The RII was considered to have increased or decreased during the study period when the interaction term was statistically significant (p < 0.05).

Absolute inequalities were measured through the Slope Index of Inequality (SII) calculated according to the following formula: SII = 2 × age-standardized mortality rate × (RII -1)/(RII+ 1). This index measures absolute differences in rates per 100,000 inhabitants between the lowest and the highest ends of the socio-economic scale and is derived from the RRI and the age- standardized overall mortality rate [8].

## Results

During the study period 31,410 men and 21,235 women died of cancer. Lung, colon, prostate, liver and stomach cancer were the most frequent causes of cancer death among men (Table 1). Among women, mortality was higher for cancer of the breast, colon, lung, stomach and pancreas (Table 2).

The trends of inequalities of total cancer mortality remained stable during the study period among men and women. Non-significant decreases of the RII may be observed: from 1.67 to 1.50 for men, from 1.15 to 1.05 for women. Decreases may also be seen for the SII, from 300.25 to 192.71 per 100,000 inhabitants among men, and from 35.61 to 10.37 per 100,000 inhabitants among women. (Tables 3 and 4).

**Table 3:** Association between mortality and educational level by cancer site and period.

	**1992–1994**			**1995–1997**			**1998–2000**			**2001–2003**			**Trend**
**Type of cancer**	**RII**	**95% CI**	**SII**	**RII**	**95% CI**	**SII**	**RII**	**95% CI**	**SII**	**RII**	**95% CI**	**SII**	**P value**
Stomach	3.05	2.12–4.40	35.87	2.97	2.06–4.29	30.09	2.72	1.86–3.99	23.17	2.93	1.99–4.31	22.30	0.98
Colon	1.25	0.94–1.65	10.93	1.09	0.84–1.41	4.23	1.01	0.79–1.28	0.50	1.05	0.82–1.34	2.18	0.71
Rectum	1.44	0.87–2.40	5.72	1.57	0.96–2.57	6.34	2.85	1.76–4.60	15.49	1.66	1.05–2.63	6.98	0.18
Larynx	2.82	1.70–4.70	15.00	3.70	2.21–6.20	17.38	4.17	2.36–7.37	14.86	3.23	1.89–5.51	12.47	0.76
Lung	2.05	1.75–2.40	110.10	1.74	1.49–2.02	83.02	2.10	1.80–2.44	101.44	1.89	1.62–2.19	81.68	0.29
Mouth and pharynx	3.37	2.15–5.28	21.26	4.85	2.97–7.93	22.22	2.24	1.42–3.54	12.29	2.98	1.80–4.94	12.54	0.14
Oesophagus	3.67	2.14–6.29	16.33	2.46	1.48–4.08	12.13	2.27	1.35–3.80	9.95	1.91	1.14–3.21	7.00	0.35
Liver	1.72	1.24–2.37	19.05	1.31	0.96–1.79	9.38	1.70	1.23–2.33	16.70	2.43	1.70–3.48	21.61	0.08
Pancreas	1.23	0.79–1.91	3.88	1.00	0.69–1.46	0.00	1.03	0.71–1.49	0.60	0.78	0.55–1.10	-5.25	0.41
Kidney	0.57	0.34–0.96	-6.58	0.96	0.54–1.71	-0.39	0.70	0.41–1.20	-3.40	1.01	0.59–1.72	0.09	0.40
Brain (central nervous system)	1.19	0.66–2.17	1.52	0.86	0.50–1.49	-1.54	0.69	0.41–1.17	-3.64	0.83	0.51–1.34	-2.11	0.59
Non-Hodgkin lymphoma	1.00	0.59–1.71	0.00	0.70	0.43–1.14	-4.65	0.82	0.51–1.32	-2.51	1.07	0.69–1.67	0.93	0.59
Leukaemia	1.34	0.81–2.21	4.54	0.87	0.55–1.39	-2.02	1.31	0.81–2.13	3.56	1.27	0.83–1.93	3.74	0.54
Gallbladder	1.58	0.65–3.82	18.33	1.16	0.52–2.60	4.74	0.64	0.3–1.37	-12.08	1.44	0.59–3.50	10.57	0.39
Prostate	1.03	0.79–1.34	1.76	1.10	0.85–1.41	5.18	1.09	0.85–1.41	3.90	0.98	0.75–1.29	-0.74	0.93
Tumours non defined	2.25	1.56–3.24	24.50	2.04	1.44–2.88	21.83	1.68	1.21–2.32	16.04	2.25	1.64–3.09	23.93	0.56

Total	1.67	1.54–1.81	300.25	1.50	1.39–1.62	224.75	1.57	1.45–1.69	231.75	1.50	1.39–1.62	192.71	0.19

Relative Index of Inequality (RII) with 95% confidence interval (CI) and Slope Index of Inequalities (SII) between the lowest and highest educational level per 100.000 inhabitants. Men aged 20 years and older. Barcelona. 1992–2003.

Trend p value: it is the p value of the comparison of the RII of the 4 periods

**Table 4:** Association between mortality and educational level by cancer site and period.

	**1992–1994**			**1995–1997**			**1998–2000**			**2001–2003**			**Trend**
**Type of cancer**	**RII**	**95% CI**	**SII**	**RII**	**95% CI**	**SII**	**RII**	**95% CI**	**SII**	**RII**	**95% CI**	**SII**	**P value**
Stomach	2.35	1.43–3.86	13.23	3.11	1.81–5.33	13.30	1.76	1.07–2.90	6.47	1.63	0.98–2.70	4.67	0.28
Colon	0.94	0.68–1.31	-1.76	1.41	1.00–1.97	8.91	1.19	0.88–1.63	4.78	1.47	1.06–2.04	8.91	0.22
Rectum	1.28	0.67–2.44	2.00	1.80	0.95–3.43	4.48	1.40	0.77–2.56	2.53	0.96	0.53–1.73	-0.28	0.55
Lung	0.66	0.42–1.06	-5.52	0.62	0.41–0.94	-6.60	0.61	0.41–0.91	-7.06	0.54	0.38–0.77	-10.36	0.91
Mouth and pharynx	0.43	0.15–1.21	-2.09	1.74	0.62–4.88	1.58	1.43	0.58–3.52	1.14	0.5	0.21–1.24	-1.57	0.09
Liver	1.57	0.94–2.61	6.22	2.48	1.46–4.21	10.95	2.16	1.26–3.68	8.45	2.27	1.33–3.85	7.95	0.63
Pancreas	0.78	0.45–1.32	-2.82	0.87	0.54–1.40	-1.66	1.09	0.69–1.73	1.07	0.86	0.55–1.32	-1.81	0.78
Kidney	0.99	0.36–2.68	-0.03	1.01	0.43–2.37	0.04	0.63	0.25–1.56	-1.30	0.78	0.34–1.79	-0.76	0.87
Brain (central nervous system)	1.39	0.69–2.81	2.00	2.20	1.11–4.39	4.84	1.57	0.81–3.07	2.87	1.17	0.63–2.19	1.05	0.57
Non-Hodgkin lymphoma	1.09	0.59–2.04	0.68	1.39	0.75–2.57	2.55	1.40	0.76–2.58	2.61	1.10	0.65–1.85	0.81	0.88
Leukaemia	0.91	0.50–1.65	-0.81	2.28	1.18–4.42	6.03	0.75	0.42–1.35	-1.97	1.07	0.64–1.79	0.59	0.07
Gallbladder	2.07	0.96–4.49	3.33	1.31	0.66–2.62	1.34	2.89	1.16–7.22	3.57	2.17	0.91–5.17	3.03	0.55
Breast	0.95	0.75–1.20	-2.66	0.94	0.73–1.20	-2.79	0.97	0.76–1.24	-1.27	0.83	0.65–1.05	-6.82	0.78
Cervix uteri	5.95	2.29–15.46	6.37	2.16	0.95–4.91	3.63	2.32	1.03–5.22	3.40	3.11	1.33–7.28	4.10	0.35
Corpus uteri	1.01	0.56–1.82	0.09	1.39	0.74–2.60	2.46	1.51	0.83–2.76	3.30	1.14	0.61–2.14	0.84	0.77
Ovary and other female genital organs	1.27	0.75–2.15	2.66	1.36	0.82–2.25	3.46	0.98	0.59–1.63	-0.20	1.12	0.71–1.79	1.23	0.81
Tumours non defined	1.27	0.81–2.00	4.07	1.29	0.83–1.99	3.96	1.16	0.75–1.79	2.00	1.54	1.01–2.35	6.14	0.81

Total	1.15	1.03–1.29	35.61	1.26	1.13–1.41	54.66	1.16	1.04–1.29	33.27	1.05	0.95–1.17	10.37	0.12

Relative Index of Inequality (RII) with 95% confidence interval (CI) and Slope Index Inequalities (SII) between the lowest and highest educational level per 100.000 inhabitants. Women aged 20 years and older. Barcelona. 1992–2003.

Trend p value: it is the p value of the comparison of the RII of the 4 periods

**Figure 1:**
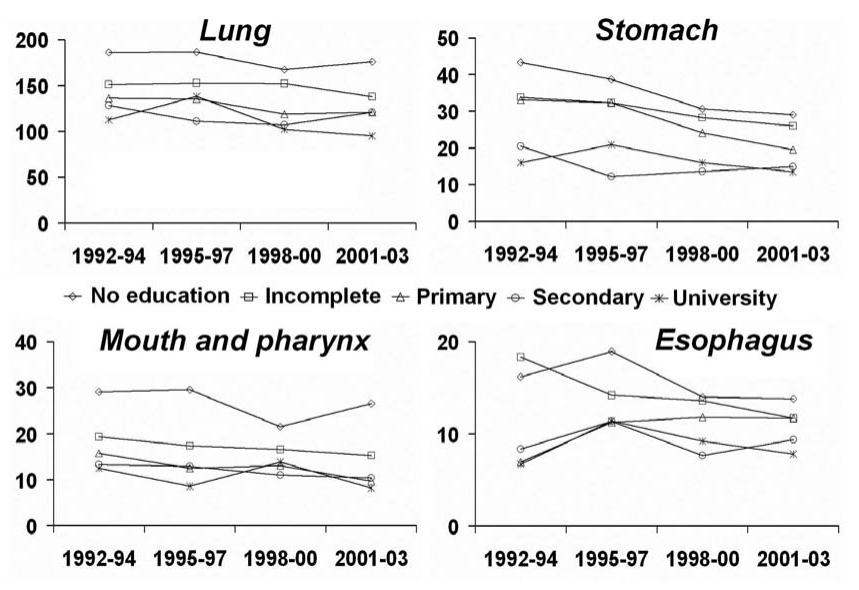
**Mortality trends for cancers of lung, stomach, mouth and pharynx and oesophagus in men (≥ 20 years) by educational level in Barcelona, 1992–2003**. Age-adjusted death rates per 100,000 inhabitants.

Among men, the trends were stable for the majority of cancer sites. Stomach, mouth and pharynx, oesophagus, larynx, and lung were the cancer sites with greater social inequalities for men (higher figures of RII and SII throughout the study period), with higher mortality among those with less education but non-significant trends (Table 3 and figure 1). Men with higher educational level had higher mortality rates for kidney cancer and melanoma but with a stable trend.

**Figure 2:**
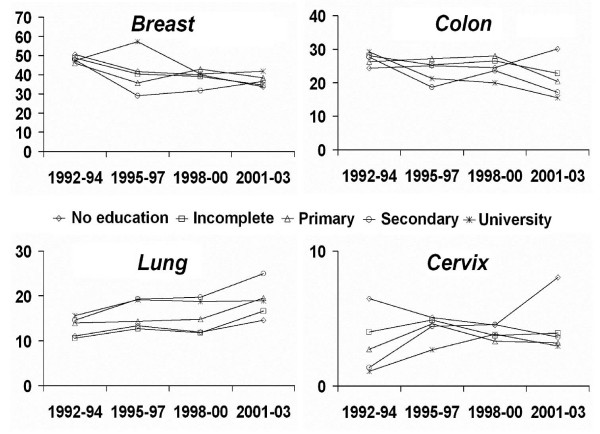
**Mortality trends for cancers of breast, colon, lung and cervix in women (≥ 20 years) by educational level in Barcelona, 1992–2003**. Age-adjusted death rates per 100,000 inhabitants.

Among women, the majority of cancer sites had a stable trend (Table 4 and figure 2). The RII for breast cancer remained near unity during the four periods, indicating the absence of a significant educational gradient of mortality. Stomach cancer presented significant inequalities among educational levels in the first three study periods, with a decrease in the SII over time, from 13.23 to 4.67 per 100,000 inhabitants. Cervix uteri cancer showed the highest education-related inequalities in all four periods (Table 4); its reduction over time was not significant. Rates of lung cancer mortality presented a steady increase throughout the study period (Figure 2) and an inverse association with schooling was found, with higher death rates among those with higher educational level (significant RII under 1 for most periods, Table 4).

## Discussion

Complementing previous studies in this population [13,23], the present study adds information for more recent periods and systematically assesses trends of inequalities associated with cancer in general, and with specific types of cancer. Therefore, the main findings of this study are the identification of those cancer types presenting the greatest inequalities between men and women in recent years, and the existence of stable trends of inequalities in the burden of this disease. Men and women with lower educational level suffered a higher burden of cancer during the whole study period. The cancer sites with larger educational inequalities were stomach, mouth and pharynx, oesophagus, larynx, and lung among men; and cervix uteri, liver, and colon among women. Mortality was higher among better-educated people for cancers of the kidney among men, and for lung cancer among women. Inequalities of cancer mortality remained stable in Barcelona during the study period except for a few cancer sites (oesophagus, pancreas prostate in men and stomach, lung, kidney, breast, cervix uteri in women) in which a slight reduction was observed.

The limited reduction in the educational gradient in cancer mortality in Barcelona needs careful consideration. Studies in other countries have also found limited reductions in inequalities of cancer mortality. In France, despite an overall reduction of mortality, inequalities increased for tumours affecting the upper respiratory tract, lung, oesophagus, and colorectal [24]. In 27 states of the US, between 1984 and 1997, increasing inequalities among employed persons were reported in lung and colorectal cancer mortality for men, and lung cancer mortality for women [25].

Inequalities in cancer mortality reflect, among other factors, the differential exposure to risk factors and different survival rates. Smoking, alcohol consumption, and dietary habits are well-known risk factors for several types of cancer, and their interactions contribute to multiply their carcinogenic effects [26]. Therefore, the study of magnitude and trends of socioeconomic inequalities in cancer outcomes should take into account the unequal exposure to these factors by persons of different socioeconomic position and sex [27-29].

For lung cancer, trends in smoking patterns explain most of the variation observed in mortality in different countries [30]. In Barcelona, smoking prevalence by social class and sex has changed in recent decades. Whereas tobacco addiction was more prevalent among more affluent men in 1983, this situation reversed after 1986, thus partially explaining the higher lung cancer mortality rates among less-schooled men. For women, however, more educated and upper social classes continue presenting a higher prevalence of smoking, although the difference has reduced in recent years [31,32]. Gender-related differences of smoking patterns are consistent with the current observation of higher lung cancer mortality among women with higher educational level in most European countries [33,34].

Poor dietary habits and their consequences, such as obesity and overweight, influence the risk of cancer in the digestive system, and can interact with tobacco and alcohol to increase the risk of other tumours, although the causal chain linking nutritional imbalances and cancer has not been fully explained [35,36]. Dietary disorders are also unequally distributed in the population [37,38], contributing to explain inequalities in mortality due to tumours of the colon, liver, stomach, oesophagus, mouth and pharynx in both genders. The infection by *Helicobacter pylori *was reported as an etiological factor of stomach cancer, which would increase the risk by several times [39]. In spite of its reduced prevalence in developed countries, this factor has been reported to affect mostly the deprived population [40-42].

Breast cancer presented an overall reduction of mortality in Barcelona, with a concurrent increase of incidence caused by screening among women [43-45]. In the current study, the relative inequality of breast cancer mortality was not significantly different from unity for any of the periods, which suggests the reversal of a previously positive socioeconomic gradient (more deaths among wealthier women). Similar changes in patterns of breast cancer mortality and incidence by educational level have been observed in New Zealand [46] and France [47].

Spain has a National Health System that ensures equal access to health services [48,49] however there are no studies based on inequalities in cancer treatment, but prevention and detection, among other factors, can play a very important role. In Barcelona a population program of breast cancer screening for women 50–69 years of age started in 1995 in the most deprived zones of the city. By 2004 it covered all city districts [50], but the highest participation rates correspond to women of less privileged social classes. The results of the Health Interview Surveys of Barcelona showed the important contribution of this population-based programme to a reduction of inequalities in breast cancer screening and also to an increase in the percentage of women having a periodic mammographic control.

In the case of cervical cancer, where the screening is opportunistic, the situation is different and the inequalities for this kind of cancer are highest, although the evidence in Barcelona shows that the relative inequality may have presented a slight decrease. However, epidemiological assessment of cervical cancer mortality is subjected to uncertainty due to a large proportion of uterine cancer being notified as "not otherwise specified"; and hence with no distinction between cancers originated in the cervix or in the corpus uteri [51].

One of the strengths of this study is the possibility to compare trends of cancer mortality by gender and educational level at the individual level. The availability of data in the Spanish context is restricted by the poor quality of socioeconomic information registered in death certificates, and by a restrictive legislation [52-54]. As a result, most studies have assessed aggregated information at the ecological level [55,56]. In Barcelona, death certificates have been linked to information gathered by local censuses since 1992, which permitted the current study to gather information at the individual level. A potential limitation of the data concerns the accuracy of death certification, which should be improved for some cancer sites [54] although a previous study done in Barcelona showed that certification quality for cancer-related causes of death was good [57]. Another limitation is the lack of data on social class based on occupational information that could greatly improve the assessment of inequalities in cancer mortality [58,59]. Notwithstanding, educational level is a relevant dimension of socioeconomic position [60]. Finally, it is worth mentioning that inequalities in mortality reflect inequalities in incidence and survival; the information necessary to study these inequalities separately is not available for Barcelona city.

## Conclusion

Cancer is a complex, multifactorial disease, and the study of cancer inequalities involves other individual and contextual factors [30]. Recent studies in the US and Europe have considered the complex interplay of factors involved in the socioeconomic gradient of cancer [61]. In especial, Krieger [62] proposed a conceptual and analytical definition of social inequalities in cancer, which spans the full cancer continuum across the life course. The present study, however, was aimed at analyzing mortality inequalities by educational level and discussing the potential effect of major risk factors for inequalities in the main cancer sites. The available data do not allow an appraisal of social and health policies, which further research should take into account.

Launched in 1985, the Europe Against Cancer programme had an ambitious target of reducing cancer mortality by 15% by the year 2000, which unfortunately was not fully achieved [63]. This programme centred the fight against the disease on the development of new technologies for prevention, diagnosis and treatment [64]. If these technologies were to be applied equally across social and educational groups, it may contribute to reduce inequalities in the burden of cancer. The accession to the European Union of countries with poorer socio-economic indices and increasing trends of cancer mortality [65,66] have made this task even more challenging. Policies aimed at reducing social inequalities in health are a worldwide demand, and their implementation must be a straightforward sign of societal commitment with human needs and rights [67,68].

## Competing interests

This study was partially financed by of the Fondo de Investigaciones Sanitarias, Instituto de Salud Carlos III (Grant n° 04/2013), CIBER Epidemiología y Salud Pública CIBERESP (Spain), Research Network in Cancer, (RTICC N° RD06/0020/0089) and the Department of Universities and Research, Government of Catalonia (AGAUR 00646).

## Authors' contributions

RP participated in designing the study, has been involved in drafting the manuscript or revising it critically for important intellectual content and has given final approval of the version to be published. CB participated in designing the study, has analysed part of the data, has been involved in drafting the manuscript or revising it critically for important intellectual content and has given final approval of the version to be published. JFA, MRS and EF have made substantial contributions to interpretation of data; have been involved in drafting the manuscript or revising it critically for important intellectual content and have given final approval of the version to be published. EA has been involved in analysing the data and has given final approval of the version to be published. MIP, GCS, MP have been involved in the design of the study, in the interpretation of the results and have given final approval of the version to be published.

## Pre-publication history

The pre-publication history for this paper can be accessed here:


